# Caffeine enhances the antidepressant-like activity of common antidepressant drugs in the forced swim test in mice

**DOI:** 10.1007/s00210-015-1189-z

**Published:** 2015-11-27

**Authors:** Aleksandra Szopa, Ewa Poleszak, Elżbieta Wyska, Anna Serefko, Sylwia Wośko, Aleksandra Wlaź, Mateusz Pieróg, Andrzej Wróbel, Piotr Wlaź

**Affiliations:** Department of Applied Pharmacy, Medical University of Lublin, Chodźki 1, PL 20-093 Lublin, Poland; Department of Pharmacokinetics and Physical Pharmacy, Collegium Medicum, Jagiellonian University, Medyczna 9, PL 30-688 Kraków, Poland; Department of Pathophysiology, Medical University of Lublin, Jaczewskiego 8, PL 20-090 Lublin, Poland; Department of Animal Physiology, Institute of Biology and Biochemistry, Faculty of Biology and Biotechnology, Maria Curie-Skłodowska University, Akademicka 19, PL 20-033 Lublin, Poland; Second Department of Gynecology, Medical University of Lublin, Jaczewskiego 8, PL 20-090 Lublin, Poland

**Keywords:** Caffeine, Antidepressants, Forced swim test, Pharmacokinetic study, Mice

## Abstract

Caffeine is the most widely used behaviorally active drug in the world which exerts its activity on central nervous system through adenosine receptors. Worrying data indicate that excessive caffeine intake applies to patients suffering from mental disorders, including depression. The main goal of the present study was to evaluate the influence of caffeine on animals’ behavior in forced swim test (FST) as well as the effect of caffeine (5 mg/kg) on the activity of six typical antidepressants, such as imipramine (15 mg/kg), desipramine (10 mg/kg), fluoxetine (5 mg/kg), paroxetine (0.5 mg/kg), escitalopram (2 mg/kg), and reboxetine (2.5 mg/kg). Locomotor activity was estimated to verify and exclude false-positive/negative results. In order to assess the influence of caffeine on the levels of antidepressant drugs studied, their concentrations were determined in murine serum and brains using high-performance liquid chromatography. The results showed that caffeine at a dose of 10, 20, and 50 mg/kg exhibited antidepressant activity in the FST, and it was not related to changes in locomotor activity in the animals. Caffeine at a dose of 5 mg/kg potentiated the activity of all antidepressants, and the observed effects were not due to the increase in locomotor activity in the animals. The interactions between caffeine and desipramine, fluoxetine, escitalopram, and reboxetine were exclusively of pharmacodynamic character, because caffeine did not cause any changes in the concentrations of these drugs neither in blood serum nor in brain tissue. As a result of joint administration of caffeine and paroxetine, an increase in the antidepressant drug concentrations in serum was observed. No such change was noticed in the brain tissue. A decrease in the antidepressant drug concentrations in brain was observed in the case of imipramine administered together with caffeine. Therefore, it can be assumed that the interactions caffeine-paroxetine and caffeine-imipramine occur at least in part in the pharmacokinetic phase.

## Introduction

Caffeine is one of the most frequently used psychoactive substances ingested mainly via food products (Fredholm et al. 1999). What is more, it is applied for therapeutic purposes when combined with analgesics (Andrews et al. 2007; Shapiro 2007) and antihistamines or in dietary supplements for weight loss (Andrews et al. 2007; Barone and Roberts 1996; Burke 2008; Shapiro 2007). Caffeine exhibits a stimulating activity which facilitates physical and mental tiredness and helps to improve thinking process (Nehlig et al. 1992). Caffeine consumption rises every year. Worrying data indicate that excessive caffeine intake applies to patients suffering from mental disorders (≥750 mg caffeine daily) (Rihs et al. 1996).

After oral administration, caffeine is absorbed from the gastrointestinal tract into the bloodstream in 99 % (Fredholm et al. 1999). The highest concentration of caffeine in plasma is reached 30–60 min after ingestion. Caffeine easily penetrates the intracellular space. It is distributed to all body fluids, that is, plasma, cerebrospinal fluid, saliva, bile, semen, milk, umbilical cord blood, and organ tissues (Arnaud 2011). Hydrophobic properties of caffeine allow its easy penetration through all biological membranes. The blood-brain barrier does not prevent caffeine (Lorist and Tops 2003). As the fraction bound to plasma proteins is low, almost all of caffeine in blood is present in a pharmacologically active form (Arnaud 2011; Lorist and Tops 2003). The main enzyme responsible for the metabolism of caffeine is the isoenzyme CYP1A2 of cytochrome P450 (Carrillo and Benitez 2000).

Caffeine is not accepted as a standard pharmacological treatment of mood disorders, but it is likely that some people can use it as an antidepressant drug in the early stages of this disease (Casas et al. 2004). It is thus possible to demonstrate the usefulness of caffeine and its derivatives in the treatment of depression. It has been shown that caffeine can reverse the monoaminergic system changes observed in depression, for example, caffeine blocking the A_1_ adenosine receptor subunit may increase the levels of catecholamines and serotonin (5-HT) in the central nervous system (CNS) (Fredholm 1995). What seems to be important is the fact that caffeine contributes to an increased 5-HT release in the limbic areas and the release of dopamine (DA) in the prefrontal cortex, which is comparable to the effect obtained with the use of antidepressants (Acquas et al. 2002; Fredholm 1995).

Caffeine exerts its activity on CNS through adenosine receptors for which it acts as an antagonist (Benowitz 1990; Dunwiddie and Masino 2001; Fredholm et al. 1999; Pettenuzzo et al. 2008). It was found in various studies, both in vitro and in vivo, that caffeine affects the activity of endogenous adenosine inhibiting the release of various neurotransmitters in CNS: acetylcholine, γ-aminobutyric acid (GABA), glutamate, DA, noradrenaline (NA), and 5-HT (El Yacoubi et al. 2003; El Yacoubi et al. 2001; Ferré et al. 1996a; Ferré et al. 1996b; Fisone et al. 2004; Williams 1987).

World Health Organization (WHO) estimated that currently, there are 350 million people around the world suffering from depressive disorders. WHO predictions indicate that by 2020, depression will be the second of civilization disease causing incapacity. Moreover, the age in which the first symptoms of depressive disorders appear is substantially reduced each year. Due to an increasing number of cases diagnosed with mental illnesses, including depression, interactions between caffeine and other drugs used in pharmacotherapy of these disorders attract more and more attention. The main goal of this study was to assess the effect of caffeine on animals’ behavior in the forced swim test (FST), which is a widely used behavioral test in estimating antidepressant properties of drugs. Moreover, we also decided to evaluate the influence of caffeine on the activity of typical antidepressant drugs, i.e., imipramine, and its metabolite desipramine—the tricyclic antidepressants (TCAs)—fluoxetine, escitalopram, and paroxetine—the selective serotonin reuptake inhibitors (SSRIs)—and reboxetine—a selective noradrenaline reuptake inhibitor (SNRI).

## Material and methods

### Animals

The experiment was carried out on naïve adult male Albino Swiss mice (25–30 g) purchased from the licensed breeder (Kolacz, Warsaw, Poland). The animals were housed in the environmentally controlled rooms with a 12-h light/dark cycle, in groups of 10 in standard cages under strictly controlled laboratory conditions—temperature maintained at 21–23 °C and relative humidity about 45–55 %. Throughout the study, the animals were given ad libitum access to water and food. The experiment began after at least 1-week acclimation period in the laboratory conditions and was conducted between 8 a.m. and 3 p.m. to minimize circadian influences. Each experimental group consisted of 7–10 animals. All procedures were conducted in accordance with the European Communities Council Directive of 22 September 2010 (2010/63/EU) and Polish legislation acts concerning animal experimentations. The experimental procedures and protocols were approved by the First Local Ethics Committee at the Medical University of Lublin. Each mouse was used only once.

### Drugs

Caffeine (1,3,7-trimethylxanthine; 5, 10, 20, and 50 mg/kg, Sigma-Aldrich, Poznań, Poland), imipramine hydrochloride (15 and 30 mg/kg, Sigma-Aldrich), desipramine hydrochloride (10 mg/kg, Sigma-Aldrich), fluoxetine hydrochloride (5 mg/kg, Sigma-Aldrich), escitalopram oxalate (2 mg/kg, Sigma-Aldrich), paroxetine hydrochloride (0.5 mg/kg, Abcam Biochemicals, Cambridge, UK), and reboxetine mesylate (2.5 mg/kg, Abcam Biochemicals) were dissolved in 0.9 % NaCl. The solutions of antidepressants were administered intraperitoneally (i.p.) 60 min before behavioral testing, whereas caffeine solution was administered i.p. 40 min before the tests. The doses and pretreatment schedules were selected on those reported in the literature and on the basis of the results of our previous experiments (David et al. 2003; Poleszak et al. 2006; Szewczyk et al. 2002; Szewczyk et al. 2009; Urani et al. 2001). All solutions were prepared immediately prior to the experiment. Animals from the control groups received i.p. injections of the vehicle (0.9 % saline). The volume of the vehicle or drug solutions for i.p. administration was 10 ml/kg.

### Forced swim test

The procedure was carried out on mice, according to the method of Porsolt et al. (1977). Each mouse was placed individually into the glass cylinders (height 25 cm, diameter 10 cm) containing 10 cm of water at 23–25 °C. The animals were left in the cylinder for 6 min. The total duration of immobility was recorded by cumulative stopwatches during the last 4 min of the 6-min-long testing period. The mouse was judged to be immobile when it ceased struggling and remained floating motionless in the water, making only the movements necessary to keep its head above the water level.

The results obtained in the forced swim test were shown as the arithmetic mean of immobility time of animals in seconds ± standard error of the mean (SEM) for each experimental group.

### Spontaneous locomotor activity

In order to avoid the risk of obtaining the false-positive/negative effects in the FST caused by a possible influence of tested agents on the locomotor activity, the spontaneous locomotor activity was measured using an animal activity meter Opto-Varimex-4 Auto-Track (Columbus Instruments, USA). This actimeter consisted of four transparent cages with a lid (43 × 43 × 32 cm), a set of four infrared emitters (each emitter has 16 laser beams), and four detectors monitoring animal movements. After i.p. pretreatment with respective drugs or drug combinations (antidepressants and saline were administered i.p. 60 min, and caffeine i.p. 40 min before the test), mice were placed individually into the cages for 10 min. Spontaneous locomotor activity was evaluated between the second and the sixth minute, which corresponds with the time interval analyzed in the FST.

The results obtained in this test were presented as the arithmetic average distance that a mouse traveled (in centimeters) ± SEM for each experimental group.

### Determination of antidepressants in serum and brain tissue

Sixty minutes following administration of the antidepressant drugs with or without caffeine, mice were decapitated to collect biological material for pharmacokinetic studies. The blood was collected into Eppendorf tubes and allowed to clot at room temperature. Subsequently, the blood was centrifuged at 10,000 rpm for 10 min and serum was collected into polyethylene tubes and frozen at −25 °C. Immediately after the decapitation, the brains were dissected from the skull, washed with 0.9 % NaCl, and also frozen at −25 °C.

Serum and brain concentrations of the studied antidepressants were assayed by a high-performance liquid chromatography (HPLC) method. The brains were homogenized in distilled water (1:4, *w*/*v*) with a tissue homogenizer TH220 (Omni International, Inc., Warrenton, VA, USA). For all studied drugs, the extraction from serum and brain homogenates was performed using the mixture of ethyl acetate/hexane (30:70, *v*/*v*). The only exceptions were paroxetine and reboxetine, for which the solvents were mixed at a 50:50 (*v*/*v*) ratio. Amitriptyline (2 μg/ml) was used as an internal standard (IS) for desipramine and imipramine, desipramine (500 ng/ml) for paroxetine and reboxetine, and paroxetine (200 ng/ml) for fluoxetine and escitalopram. In order to isolate imipramine and desipramine, to serum (200 μl) and brain homogenate (0.5 ml) containing these drugs, the IS was added and the samples were alkalized with 100 and 250 μl of 4 M NaOH, respectively. Then, the samples were extracted with 5 ml of the extraction reagent by shaking for 20 min (IKA Vibrax VXR, Germany). After centrifugation at 3000 rpm for 20 min (Universal 32, Hettich, Germany), the organic layer was transferred to a new tube containing a 200 μl solution of 0.1 M H_2_SO_4_ and methanol (90:10, *v*/*v*), shaken for 0.5 h and then centrifuged for 15 min (3000 rpm). A 50-μl aliquot of this solution was injected into the HPLC system. In the case of escitalopram, the procedure was similar with the exception that the extraction with an organic reagent (3 ml) was repeated two times, 1 ml of brain homogenate was used, and the volume of the acidic phase was 100 μl. Fluoxetine, paroxetine, and reboxetine were extracted from 200 μl of serum after addition of the appropriate IS, 100 μl of 4 M NaOH, and 3 ml of the extraction reagent. The procedure was repeated twice. In turn, to 1 ml of brain homogenates containing these drugs, the IS was added and the samples were alkalized with 500 μl of 4 M NaOH. After the addition of 1 ml of the concentrated NaCl solution (10 g/50 ml), the samples were vortexed for 15 s and 5 ml of the extraction reagent was added. Then, serum samples and brain homogenates were shaken for 20 min and centrifuged for 15 min at 3000 rpm. After the centrifugation, the organic layer was transferred into a conical glass tube and evaporated to dryness at 37 °C under a gentle stream of nitrogen in a water bath. The residue was dissolved with 100 μl of methanol, and 50 μl of this solution was injected into the HPLC system.

The HPLC system (Thermo Separation Products, San Jose, CA, USA) consisted of a P100 isocratic pump, a UV100 variable-wavelength UV/VIS detector, a Rheodyne 7125 injector (Rheodyne, Cotati, CA, USA) with a 50-μl sample loop, and a Chromjet SP4400 computing integrator.

All analyses were performed on a 250 × 4 mm LiChrospher^®^100 RP-18 column with a particle size of 5 μm (Merck, Darmstadt, Germany) protected with a guard column (4 × 4 mm) with the same packing material. The mobile phase consisting of acetonitrile and 50 mM potassium dihydrogen phosphate was mixed at a ratio of 60:40 (*v*/*v*) for imipramine, desipramine, and fluoxetine and 65:35 (*v*/*v*) for escitalopram, paroxetine, and reboxetine and run at 1 ml/min. Chromatographic analysis was carried out at 21 °C, and the analytical wavelength of 227 nm for fluoxetine, 240 nm for escitalopram, and 214 nm for the remaining antidepressants studied.

The calibration curves constructed by plotting the ratio of the peak heights of the studied drug to IS versus concentration of the drug were linear in the tested concentration ranges. No interfering peaks were observed in the chromatograms. The assays were reproducible with low intra- and inter-day variation (coefficient of variation less than 10 %). The extraction efficiencies of the analyzed compounds and internal standards ranged from 66 to 97 %. Antidepressant concentrations were expressed in nanograms per milliliter of serum or nanograms per gram of wet brain tissue.

### Statistical analysis

The statistical analysis of the results obtained in the FST and the locomotor activity assessment following caffeine administration was carried out using one-way ANOVA with Dunnett’s post hoc test and after joint treatments using two-way ANOVA with Bonferroni’s post hoc test. Interactions occurring between tested agents were analyzed with one-way ANOVA also with Bonferroni’s post hoc test. The concentrations of the tested antidepressant drugs in blood and brains of mice in the presence and absence of caffeine were compared using Student’s *t* test. *p* values less than or equal to 0.05 were considered statistically significant.

## Results

### Caffeine dose-effect relationship in the FST

**Fig. 1:**
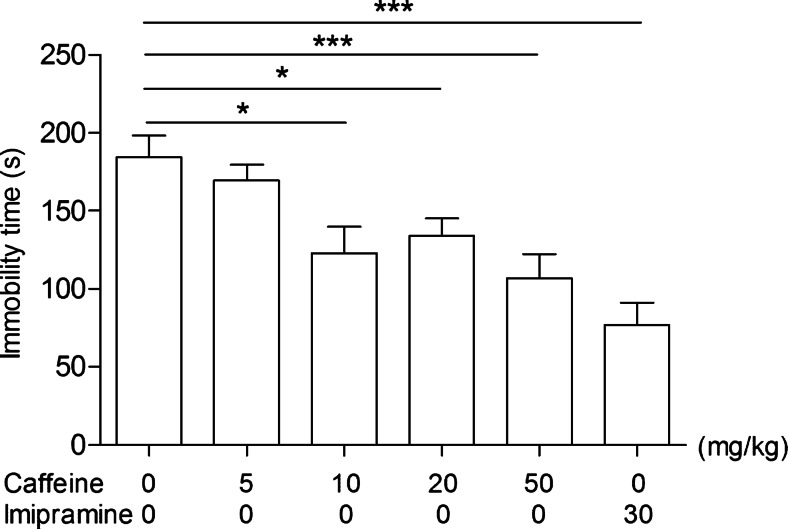
The antidepressant activity of caffeine in the forced swim test in mice. Caffeine and saline were administered i.p. 40 min, and imipramine i.p. 60 min before the test. The data are presented as the means ± SEM. Each experimental group consisted of 10 animals. *p < 0.05, ***p < 0.001 (one-way ANOVA followed by Dunnett’s post hoc test)

In order to determine its antidepressant activity, caffeine was used in the following doses: 5, 10, 20, and 50 mg/kg (Fig. 1) [one-way ANOVA: *F*(5,55) = 8.536; *p* < 0.0001]. Statistical analysis of the results showed that caffeine used at a dose of 5 mg/kg had no statistically significant effect (*p* > 0.05) on the reduction of the immobility time in mice. However, caffeine administered at a dose of 10, 20, and 50 mg/kg significantly reduced the total time of immobility in comparison with the control group.

### The influence of caffeine on the antidepressant activity of tested drugs in the FST

#### Effect of combined administration of caffeine and imipramine in the FST

**Fig. 2:**
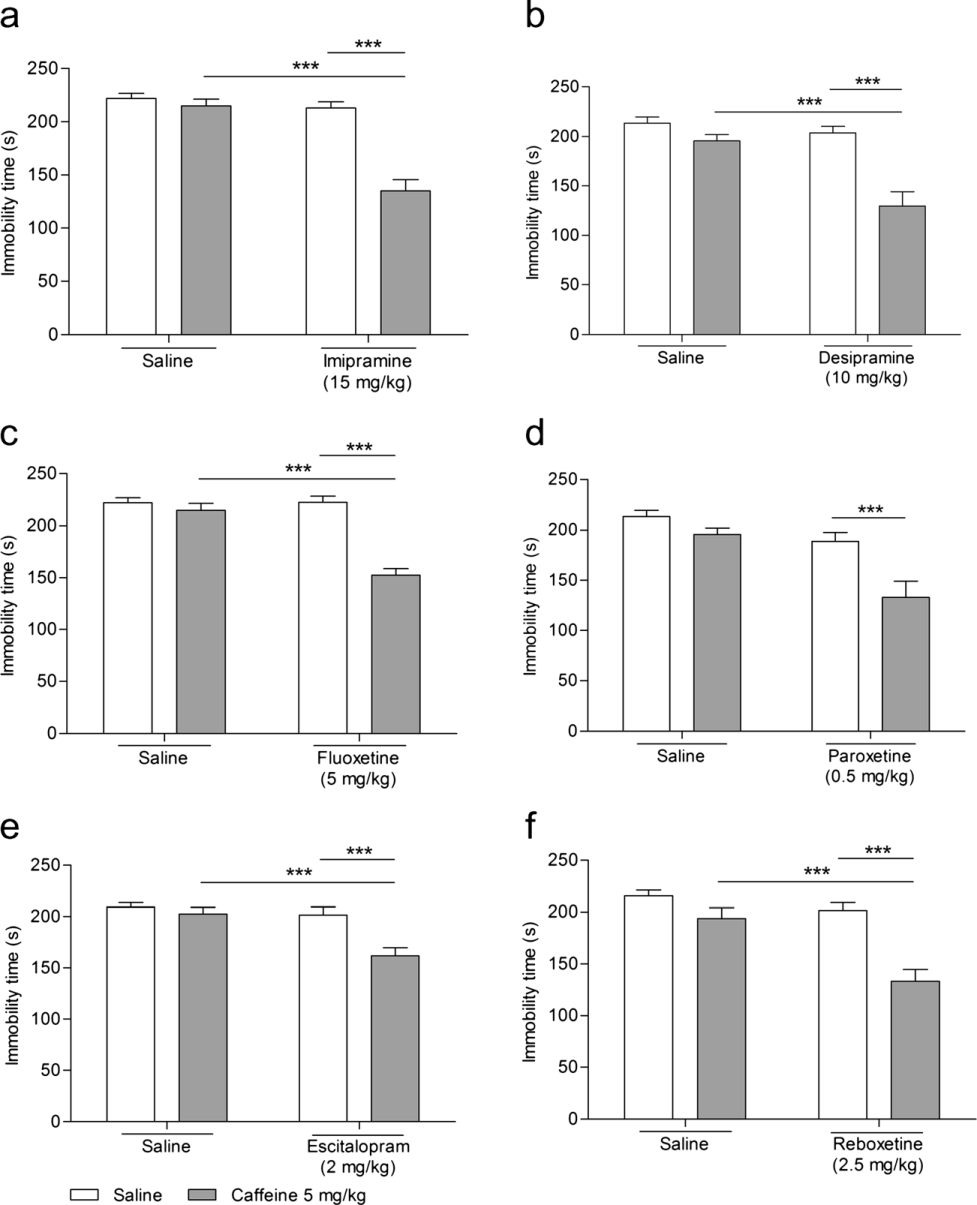
Effect of combined administration of caffeine and antidepressants in the forced swim test in mice. Caffeine was administered i.p. 40 min, and all antidepressants and saline were administered i.p. 60 min before the test. The values represent mean ± SEM. (*n* = 9–10 per group). ***p < 0.001 (two-way ANOVA followed by Bonferroni’s post hoc test). The control groups for imipramine and fluoxetine as well as desipramine and paroxetine are the same

The effect of the combined administration of caffeine and imipramine on the total duration of the immobility time in mice is shown in Fig. 2a. Caffeine (5 mg/kg) injected in combination with imipramine (15 mg/kg) significantly reduced the immobility time in the FST in mice (Fig. 2a). We carried out analysis using two-way ANOVA followed by one-way ANOVA with Bonferroni’s post hoc test.

Two-way ANOVA demonstrated an interaction between imipramine and caffeine [*F*(1,28) = 24.86, *p* < 0.0001]. One-way ANOVAs followed by Bonferroni’s post hoc test showed no effect of caffeine (*p* > 0.05) and no effect of imipramine (*p* > 0.05) alone. However, the effect of imipramine became significant in the presence of caffeine (*p* < 0.0001).

#### Effect of combined administration of caffeine and desipramine in the FST

The effect of the combined administration of caffeine and desipramine on the total duration of the immobility time in mice is shown in Fig. 2b. Caffeine (5 mg/kg) injected in combination with desipramine (10 mg/kg) significantly reduced the immobility time in the FST in mice (Fig. 2b). We carried out analysis using two-way ANOVA followed by one-way ANOVA with Bonferroni’s post hoc test.

Two-way ANOVA demonstrated an interaction between desipramine and caffeine [*F*(1,35) = 9.06, *p* < 0.0048]. One-way ANOVAs followed by Bonferroni’s post hoc test showed no effect of caffeine (*p* > 0.05) and no effect of desipramine (*p* > 0.05) alone. However, the effect of desipramine became significant in the presence of caffeine (*p* < 0.0001).

#### Effect of combined administration of caffeine and fluoxetine in the FST

The effect of the combined administration of caffeine and fluoxetine on the total duration of the immobility time in mice is shown in Fig. 2c. Caffeine (5 mg/kg) injected in combination with fluoxetine (5 mg/kg) significantly reduced the immobility time in the FST in mice (Fig. 2c). We carried out analysis using two-way ANOVA followed by one-way ANOVA with Bonferroni’s post hoc test.

Two-way ANOVA demonstrated an interaction between fluoxetine and caffeine [*F*(1,28) = 29.10, *p* < 0.0001]. One-way ANOVAs followed by Bonferroni’s post hoc test showed no effect of caffeine (*p* > 0.05) and no effect of fluoxetine (*p* > 0.05) alone. However, the effect of fluoxetine became significant in the presence of caffeine (*p* < 0.0001).

#### Effect of combined administration of caffeine and paroxetine in the FST

The effect of the combined administration of caffeine and paroxetine on the total duration of the immobility time in mice is shown in Fig. 2d. Two-way ANOVA demonstrated a lack of interaction between paroxetine and caffeine [*F*(1,36) = 3.45, *p* = 0.0716], but a significant effect of paroxetine [*F*(1,36) = 18.43, *p* = 0.0001] and a significant effect of caffeine [*F*(1,36) = 13.07, *p* = 0.0009].

#### Effect of combined administration of caffeine and escitalopram in the FST

The effect of the combined administration of caffeine and escitalopram on the total duration of the immobility time in mice is shown in Fig. 2e. Caffeine (5 mg/kg) injected in combination with escitalopram (2 mg/kg) significantly reduced the immobility time in the FST in mice (Fig. 2e). We carried out analysis using two-way ANOVA followed by one-way ANOVA with Bonferroni’s post hoc test.

Two-way ANOVA demonstrated an interaction between escitalopram and caffeine [*F*(1,36) = 5.88, *p* = 0.0204]. One-way ANOVAs followed by Bonferroni’s post hoc test showed no effect of caffeine (*p* > 0.05) and no effect of escitalopram (*p* > 0.05) alone. However, the effect of escitalopram became significant in the presence of caffeine (*p* < 0.0001).

#### Effect of combined administration of caffeine and reboxetine in the FST

The effect of the combined administration of caffeine and reboxetine on the total duration of the immobility time in mice is shown in Fig. 2f. Caffeine (5 mg/kg) injected in combination with reboxetine (2.5 mg/kg) significantly reduced the immobility time in the FST in mice (Fig. 2f). We carried out analysis using two-way ANOVA followed by one-way ANOVA with Bonferroni’s post hoc test.

Two-way ANOVA demonstrated an interaction between reboxetine and caffeine [*F*(1,35) = 6.96, *p* = 0.0124]. One-way ANOVAs followed by Bonferroni’s post hoc test showed no effect of caffeine (*p* > 0.05) and no effect of reboxetine (*p* > 0.05) alone. However, the effect of reboxetine became significant in the presence of caffeine (*p* < 0.0001).

### Effect of caffeine on locomotor activity in mice

The effect of caffeine (5, 10, 20, and 50 mg/kg) on spontaneous locomotor activity in mice is shown in Table 1 [one-way ANOVA, *F*(4,35) = 0.7440, *p* = 0.5686]. Statistical analysis of the results showed that caffeine used in all tested doses had no statistically significant effect on locomotor activity in mice versus control group.

**Table 1:** Effect of caffeine on locomotor activity in mice

Treatment (mg/kg)	Distance traveled (cm)
Saline (control group)	906.1 ± 65.89
Caffeine 5	1087.0 ± 53.00
Caffeine 10	1158.0 ± 153.70
Caffeine 25	1144.0 ± 136.20
Caffeine 50	1074.0 ± 93.82

Caffeine and saline were administered i.p. 40 min before the test. Distance traveled was recorded between the second and the sixth minute of the test. The data are presented as the means ± SEM. Each experimental group consisted of 7–8 animals. The results were considered statistically significant if *p* < 0.05 (one-way ANOVA followed by Dunnett’s post hoc test)

### Effect of combined administration of caffeine and antidepressants on locomotor activity in mice

The effect of the combined administration of caffeine and tested antidepressant drugs on spontaneous locomotor activity in mice is shown in Table 2. Caffeine or antidepressants (imipramine, fluoxetine, paroxetine, reboxetine, and escitalopram) administered either alone or combined together had no statistically significant effects on the locomotor activity in mice (Table 2). Desipramine administered alone had no significant effects on the locomotor activity in mice, while co-administered with caffeine decreased the locomotor activity in mice in comparison with the control and caffeine-treated group [one-way ANOVA, *F*(3,28) = 7.309, *p* < 0.001 and *p* < 0.01, respectively] (Table 2).

**Table 2:** Effect of treatments on spontaneous locomotor activity in mice

	Treatment (mg/kg)	Distance traveled (cm)
(A)	Saline + saline	637.6 ± 53.12
	Caffeine 5 + saline	663.9 ± 35.96
	Imipramine 15 + saline	621.4 ± 77.17
	Caffeine 5 + imipramine 15	558.6 ± 48.21
	Fluoxetine 5 + saline	628.4 ± 64.80
	Caffeine 5 + fluoxetine 5	643.7 ± 95.11
(B)	Saline + saline	746.5 ± 85.47
	Caffeine 5 + saline	910.1 ± 76.54
	Desipramine 10 + saline	520.3 ± 67.67
	Caffeine 5 + desipramine 10	539.8 ± 31.14 ^^
	Paroxetine 0.5 + saline	759.0 ± 97.15
	Caffeine 5 + paroxetine 0.5	967.3 ± 124.6
(C)	Saline + saline	674.5 ± 56.72
	Caffeine 5 + saline	815.3 ± 57.72
	Escitalopram 2 + saline	794.5 ± 75.21
	Caffeine 5 + escitalopram 2	802.0 ± 73.10
(D)	Saline + saline	615.1 ± 37.42
	Caffeine 5 + saline	800.0 ± 32.97
	Reboxetine 2.5 + saline	505.3 ± 78.64
	Caffeine 5 + reboxetine 2.5	696.0 ± 68.45

Antidepressants and saline were administered i.p. 60 min, and caffeine i.p. 40 min before the experiment. Distance traveled was recorded between the second and the sixth minute of the test. Each experimental group consisted of 7–8 animals. Data are presented as the means ± SEM

^^*p* < 0.01 versus caffeine-treated group (two-way ANOVA followed by Bonferroni’s post hoc test)

Two-way ANOVA demonstrated:

(A) No effect of imipramine [*F*(1,25) = 1.19, *p* = 0.2857], no effect of caffeine [*F*(1,25) = 0.11, *p* = 0.7450], and no interaction [*F*(1,25) = 0.64, *p* = 0.4312].
(B) Extremely significant effect of desipramine [*F*(1,28) = 19.02, *p* = 0.0002], no effect of caffeine [*F*(1,28) = 1.79, *p* = 0.1914], and no interaction [*F*(1,28) = 1.11, *p* = 0.3011].
(C) No effect of fluoxetine [*F*(1,26) = 0.05, *p* = 0.8237], no effect of caffeine [*F*(1,26) = 0.10, *p* = 0.7527], and no interaction [*F*(1,26) = 0.01, *p* = 0.9342].
(D) No effect of paroxetine [*F*(1,28) = 0.13, *p* = 0.7241], no quite significant effect of caffeine [*F*(1,28) = 3.63, *p* = 0.0672], and no interaction [*F*(1,28) = 0.05, *p* = 0.8209].
(E) No effect of escitalopram [*F*(1,28) = 0.65, *p* = 0.4271], no effect of caffeine [*F*(1,28) = 1.25, *p* = 0.2726], and no interaction [*F*(1,28) = 1.1, *p* = 0.3231].
(F) Very significant effect of reboxetine [*F*(1,23) = 9.95, *p* = 0.0044], not quite significant effect of caffeine [*F*(1,23) = 3.23, *p* = 0.0857], and no interaction [*F*(1,23) = 0.00, *p* = 0.9612].

### Pharmacokinetic studies

The effect of caffeine on serum and brain concentrations of the tested antidepressants in mice is shown in Tables 3 and 4. Only in the case of co-administration of caffeine and paroxetine an increased paroxetine concentration in serum was observed (*t* test, *p* = 0.0296) without affecting its concentration in the brain tissue. Conversely, caffeine lowered the concentration of imipramine in brain tissue (*t* test, *p* = 0.0207) with no significant effect on its concentration in serum (Tables 3 and 4).

**Table 3:** Effect of caffeine on the concentrations of antidepressants in mouse serum

	Treatment	Drug concentration (ng/ml)
(A)	Imipramine 15 + saline	352.4 ± 72.13
	(Metabolite–desipramine)	(34.69 ± 6.17)
	Imipramine 15 + caffeine 5	213.8 ± 31.27
	(Metabolite–desipramine)	(43.92 ± 11.83)
(B)	Desipramine 10 + saline	203.7 ± 22.54
	Desipramine 10 + caffeine 5	220.6 ± 25.05
(C)	Fluoxetine 5 + saline	404.5 ± 41.65
	Fluoxetine 5 + caffeine 5	435.0 ± 36.87
(D)	Paroxetine 0.5 + saline	35.7 ± 5.68
	Paroxetine 0.5 + caffeine 5	63.9 ± 10.50*
(E)	Escitalopram 2 + saline	81.1 ± 3.40
	Escitalopram 2 + caffeine 5	86.4 ± 5.15
(F)	Reboxetine 2.5 + saline	211.0 ± 23.05
	Reboxetine 2.5 + caffeine 5	160.4 ± 15.42

Antidepressants were administered i.p. 60 min, and caffeine 40 min before decapitation. Each experimental group consisted of 9–10 animals. Results are presented as mean values ± SEM

*p < 0.05 compared to the respective control group (Student’s *t* test)

**Table 4:** Effect of caffeine on the concentrations of antidepressants in mouse brain

	Treatment	Drug concentration (ng/g)
(A)	Imipramine 15 + saline	5022.0 ± 749.90
	(Metabolite–desipramine)	(433.0 ± 100.10)
	Imipramine 15 + caffeine 5	2920.0 ± 354.00*
	(Metabolite–desipramine)	(320.0 ± 62.09)
(B)	Desipramine 10 + saline	2100.0 ± 201.30
	Desipramine + caffeine 5	2455.0 ± 259.40
(C)	Fluoxetine 5 + saline	6091.0 ± 484.90
	Fluoxetine 5 + caffeine 5	6915.0 ± 268.10
(D)	Paroxetine 0.5 + saline	129.5 ± 9.78
	Paroxetine 0.5 + caffeine 5	144.8 ± 15.92
(E)	Escitalopram 2 + saline	372.5 ± 29.12
	Escitalopram 2 + caffeine 5	371.2 ± 31.17
(F)	Reboxetine 2.5 + saline	411.7 ± 31.94
	Reboxetine 2.5 + caffeine 5	378.8 ± 34.06

Antidepressants were administered i.p. 60 min, and caffeine 40 min before decapitation. Each experimental group consisted of 10 animals. Results are presented as mean values ± SEM

*p < 0.05 compared to the respective control group (Student’s *t* test)

## Discussion

Caffeine is one of the most commonly used psychoactive substances in the world (Solinas et al. 2002). Products containing caffeine have gained popularity due to the stimulant effect on the CNS. An excessive intake of caffeine is particularly prevalent amongst the patients hospitalized due to mental disorders. It is estimated that as many as 22 % of these people consumed more than 750 mg caffeine a day, whereas such a high intake of this methylxanthine was found in 9 % of the general population (Hughes et al. 1998). Caffeine acts on the CNS both directly and indirectly. In non-toxic doses, caffeine acts mainly as an antagonist of adenosine inhibitory A_1_ and stimulatory A_2A_ receptors and these receptors control the neuronal excitability and the release of several neurotransmitters, including ACh, DA, NA, and 5-HT. Only in higher doses caffeine is capable to inhibit the activity of phosphodiesterases and GABA receptors or mobilize intracellular Ca^2+^ (Fredholm 1995; Goldberg et al. 1982; Lorist and Tops 2003; Williams 1987).

Numerous animal studies indicate that psychostimulant-active compounds such as caffeine or amphetamine reduce the duration of immobility time in the FST (Enríquez-Castillo et al. 2008; Gan et al. 2009; Vieira et al. 2008), and the observed effect is comparable with the one recorded after administration of tricyclic antidepressants (i.e., imipramine, desipramine), SSRIs (i.e., fluoxetine, paroxetine, or sertraline), and SNRIs (Page et al. 1999). Other adenosine receptor antagonists, e.g., istradefylline (KW 6002) (El Yacoubi et al. 2003; El Yacoubi et al. 2001; Yamada et al. 2013), and SCH 58261 (El Yacoubi et al. 2003; El Yacoubi et al. 2001) exerted an antidepressant-like activity in the FST and the tail suspension test (TST), as well.

However, according to the literature (Batalha et al. 2013; Pechlivanova et al. 2012) (Lucas et al. 2011; Smith 2002), caffeine affects mood of animals and humans in a dose-dependent manner. A low dose of this methylxanthine (10 mg/kg) reduces the duration of immobility time of rodents in the FST, while its high dose (100 mg/kg) produces the opposite effect (Gan et al. 2009). Also, El Yacoubi et al. showed that caffeine at stimulant doses was effective in FST (El Yacoubi et al. 2003). It is believed that the influence on catecholamines and 5-HT neurotransmission is responsible for the response observed after administration of the low dose of caffeine (Rénéric and Lucki 1998). An injection of the higher dose is probably related to an excessive increase in the level of ACh and 5-HT in the CNS, which leads to the inhibition of adenosine receptors (Nikodijević et al. 1993a; Nikodijević et al. 1993b). What is more, in their most recent research, Kaster et al. showed protective effects of caffeine on maladaptive changes in the model of chronic unpredictable stress, which was comparable with the selective blocking of adenosine A_2A_ receptors. This study pointed out that adenosine A_2A_ receptor blockers have therapeutic benefits and their use should be considered as a novel therapy to counteract the negative impact of chronic stress on mood (Kaster et al. 2015). The results of our study confirmed the antidepressant-like activity of caffeine given at the doses of 10, 20, and 50 mg/kg. Moreover, the highest dose used by us exerted an effect similar to the action of imipramine administered at an active dose (30 mg/kg).

We also demonstrated that caffeine may affect the action of typical antidepressant drugs, such as imipramine, desipramine, fluoxetine, escitalopram, paroxetine, and reboxetine. Co-administration of caffeine with these agents at the non-active doses resulted in a statistically significant reduction in the immobility times in the FST compared with the control groups. It should be noted that the observed effects were not due to the increase in locomotor activity of animals. Most probably, the shorter immobility time noted after the joint administration of caffeine and imipramine or desipramine may be a consequence of the enhancement of NA and 5-HT transduction (Lorden and Nunn 1982). Caffeine influences A_1_ receptors, which are located on serotonergic neurons in the dorsal nucleus structures (Mössner et al. 2000) and in the locus coeruleus (Regenold and Illes 1990). It is known that the stimulation by endogenous adenosine or adenosine selective agonists causes a reduction in the activity of serotonergic neurons (Regenold and Illes 1990), which leads to a decrease in the adrenergic and serotonergic transmission (Okada et al. 2002). Caffeine through antagonism of the A_1_ receptors, inter alia, blocks the effects of endogenous adenosine and increases NA and 5-HT transduction in the CNS (Fredholm et al. 1999; Kulkarni and Mehta 1985; Schlosberg 1984), which may explain the synergistic effect on the performance of the tested TCAs. Another cause of the synergistic effect of caffeine and imipramine or desipramine may be an impaired metabolism of caffeine by these antidepressants. It has been shown that both imipramine and desipramine inhibit the demethylation and hydroxylation of caffeine which reduces the elimination of caffeine from the organism (Daniel et al. 2003). In the research published by Robles-Molina (2012), the concurrent administration of caffeine and desipramine did not change the locomotor activity of animals, while we noticed their hypolocomotion. However, such a discrepancy might arise due to the application of different doses of the tested substances (0.31, 1 mg/kg of caffeine and 0.31, 1, 3.1 mg/kg of desipramine versus 5 mg/kg of caffeine and 10 mg/kg of desipramine) and different strains of mice (BALB/c versus Albino Swiss).

As demonstrated in the present study, caffeine augmented the activity of the SSRI agents, which is a group of drugs that are currently the first choice in the treatment of depressive disorders. They are characterized by a safer profile of adverse reactions compared with other antidepressants, and consequently, they are better tolerated by patients. The recorded synergy can also be explained by the inhibitory effect of caffeine on the effects of endogenous adenosine, which decreases the release of many neurotransmitters in the CNS, including 5-HT (Mössner et al. 2000). This blockage leads to an increase in the amount of 5-HT in the synapse and modulation of the serotoninergic transmission. Our observations are consistent with the previous reports on this subject. For example, it was shown that the administration of caffeine in the diet of rats causes an increase in the concentration of 5-HT, 5-hydroxyindoleacetic acid (a breakdown product of 5-HT), and tryptophan (the starting material for the production of 5-HT) in the CNS (Yokogoshi et al. 1987). A similar effect was observed in rats after the administration of caffeine i.p. at doses of 10–100 mg/kg (Fernstrom et al. 1984; Haleem et al. 1995). In vitro studies have also shown increased levels of 5-HT in biological material (brain stem, brain, and cerebellum) collected from rats after administration of caffeine (Berkowitz and Spector 1971; Stromberg and Waldeck 1973).

As previously mentioned, caffeine is supposed to enhance a release of NA in the CNS (Williams 1987). However, the research involving methylxanthines (including caffeine) showed conflicting results in terms of their impact on the level of catecholamines. On the one hand, it has been demonstrated that methylxanthines do not affect the concentration of NA in the CNS of rats (Karasawa et al. 1976; Schlosberg 1984); on the other hand, it has been reported that they stimulate the metabolism and the release of catecholamines in the brain (Atkinson and Enslen 1976; Costill et al. 1978). The enhancement of anti-immobility action of reboxetine by caffeine co-administration observed in our study is probably related to an increased amount of NA in the CNS. As mentioned before, reboxetine blocks neuronal reuptake of NA and the level of this neurotransmitter rises in the CNS. Caffeine, due to antagonism of adenosine receptors, also increases the release of NA, which has been observed in preclinical studies and clinical trials; after administration of single doses of caffeine, increased levels of NA were observed in blood and urine in humans (Benowitz 1990; Papadelis et al. 2003) and in blood and brains of laboratory animals (Bender and Bobrova 1982; Goldberg et al. 1982).

Hydrophobic properties of caffeine allow its easy penetration through all biological membranes. It is distributed to all body fluids—plasma, cerebrospinal fluid, saliva, bile, semen, milk, umbilical cord blood, and organ tissues (Arnaud 2011). Almost all of caffeine is present in a pharmacologically active form, since the fraction bound to plasma proteins is relatively small (McCall et al. 1982). Pharmacokinetic studies carried out in the present research were aimed at assessing concentrations of antidepressants in blood and brain of mice after their combined administration with caffeine and estimating the nature of the agent-agent interactions. The present work is also the first in which such an attempt was made. The existing data on changes in the therapeutic effect of antidepressants caused by concomitant administration of caffeine were based solely on the results obtained in behavioral tests (Kale and Addepalli 2014; Robles-Molina et al. 2012). The available literature emphasizes the role of CYP1A2, the main enzyme responsible for the metabolism of caffeine, in the metabolism of drugs from different therapeutic groups, including antidepressants (e.g., imipramine, mianserin, amitriptyline, or fluvoxamine). Clinically significant pharmacokinetic interactions between caffeine and these substances is related to the amount and activity of this isoenzyme (Lin and Lu 1998; Pelkonen et al. 1998). We found that caffeine does not affect the concentrations of most of the tested antidepressants (desipramine, fluoxetine, escitalopram, reboxetine) either in serum or brain tissue. These results suggest that interactions observed between caffeine and these drugs presumably are pharmacodynamic, which refers to changes at the cellular level. We reported a statistically significant reduction in the amount of imipramine in brain tissue, with no statistically significant changes in serum drug concentration after a concurrent administration with caffeine. It is possible that the observed alterations were associated with the modifications in the biotransformation of imipramine, since both caffeine and imipramine are metabolized by the same isoenzyme of cytochrome P450 (Carrillo and Benitez 2000) or imipramine transport through blood-brain barrier. The interplay between caffeine and paroxetine demonstrated in the course of our study is not entirely clear. As a result of the joint administration of these agents, there has been an increase in the serum concentration of paroxetine, but concentration changes in the brain, which is the target site of action of antidepressants, have not been observed. In the case of drugs acting on the CNS, the concentration of drug in the brain structures is responsible for the pharmacological effect. However, the question of the correlation between the drug concentration in blood and CNS is controversial. It is believed that changes in the serum level should reflect changes occurring in the amount of drug in the brain tissue. No such relationship may be due to the delay in the transport of the drug through the blood-brain barrier (Burke and Preskorn 2004). Accordingly, it can be concluded that the interaction between caffeine and paroxetine takes place in the pharmacokinetic phase. Both observed pharmacokinetic interactions could have important consequences for patients that are coffee drinkers or otherwise caffeine users under imipramine or paroxetine therapy.

## Conclusions

In summary, we confirmed that caffeine produced an antidepressant-like effect and demonstrated a caffeine-induced enhancement of the antidepressant effect of imipramine, desipramine, fluoxetine, paroxetine, escitalopram, and reboxetine in the Porsolt’s test without stimulation of locomotor activity. The interplay between the tested methylxanthine and desipramine, fluoxetine, escitalopram, and reboxetine seems to be exclusively pharmacodynamic in nature, whereas an increased antidepressant activity of paroxetine or imipramine was at least partly related to their pharmacokinetic interaction with caffeine. However, when formulating conclusions concerning the type of the observed interactions on the basis of the pharmacokinetic studies, one should be particularly careful, since biotransformation of drugs may proceed differently in animals and humans.
